# Comparative analysis of cystic echinococcosis burden trends: a systematic evaluation of global and Chinese regional patterns using global burden of disease study 2021 data

**DOI:** 10.1186/s13071-025-07214-y

**Published:** 2025-12-28

**Authors:** Weiwei Xiao, Xiaoshen Liu, Lei Du, Yuan Tian, Chenxing Li, Li Ren

**Affiliations:** 1 Department of Hepatobiliary and Pancreatic Surgery, School of Clinical Medicine, Affiliated Hospital of Qinghai University, Qinghai University, Xining, 810001 Qinghai China; 2 Critical Care Medicine, School of Clinical Medicine, Affiliated Hospital of Qinghai University, Qinghai University, Xining, 810001 Qinghai China; 3 Qinghai Research Key Laboratory for Echinococcosis, Xining, 810001 Qinghai China

**Keywords:** Cystic echinococcosis, Incidence, Prevalence, Mortality, Disability-adjusted life years, Global burden of disease

## Abstract

**Background:**

This study leveraged the Global Burden of Disease (GBD) 2021 database to comprehensively evaluate the trends in the disease burden of cystic echinococcosis (CE) in China from 1990 to 2021, situating its unique trajectory within the global context to inform targeted control strategies.

**Methods:**

Based on data from the GBD 2021 study, the incidence, prevalence, mortality, and disability-adjusted life years (DALYs) of CE were analyzed. Joinpoint regression was applied to calculate the average annual percentage change (AAPC), decomposition analysis was conducted to identify key driving factors, and frontier analysis was used to assess reduction potential. Subgroup analyses were stratified by age, sex, and region.

**Results:**

Globally, the age-standardized incidence rate (ASIR) and age-standardized prevalence rate (ASPR) of CE remained relatively stable from 1990 to 2021. In contrast, China experienced sharp increases in ASIR (AAPC = 2.94%) and ASPR (AAPC = 3.13%). Age-standardized mortality rate (ASMR) and age-standardized DALY rate (ASDR) declined globally and in China, though China’s ASDR reduction (AAPC = −2.71%) lagged behind the global rate (AAPC = −4.0%). Decomposition analysis indicated that epidemiological deterioration was the primary driver of increased cases in China, while healthcare improvements contributed to reduced deaths and DALYs. Females had higher incidence and prevalence, particularly among those aged over 35, whereas males exhibited higher mortality and DALYs. The global CE burden was negatively correlated with the Sociodemographic Index (SDI). Although China approached the efficiency frontier in disease control, elevated ASPR and ASDR indicated persistent transmission and latent infections, suggesting further reduction potential.

**Conclusions:**

China faces rising CE incidence and prevalence despite improved outcomes, owing to delayed diagnosis and unbalanced resources. Aging and persistent exposure have worsened the burden, especially among middle-aged adults and females. Strategic priorities include enhanced prevention in the elderly, improved screening for women, intensified management of severe male cases, and balanced treatment/prevention approaches. SDI is a key determinant of CE burden, requiring focused interventions in low-SDI regions. Targeted monitoring of ASPR and ASDR is crucial to reduce the impact of historical transmission and achieve World Health Organization (WHO) targets.

**Graphical Abstract:**

**Figure:**
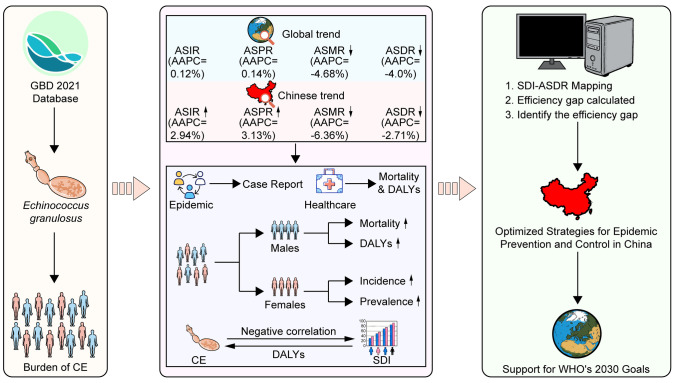

## Background

Echinococcosis, a globally prevalent zoonotic parasitic disease caused by larval infection of the genus *Echinococcus*, poses significant threats to human health, with cystic echinococcosis (CE) being the most common manifestation [1]. The World Health Organization (WHO) has classified CE as a neglected tropical disease (NTD) and incorporated it into the 2021–2030 roadmap for NTD prevention and control [2]. Concurrently, a joint report by the United Nations Food and Agriculture Organization (FAO) has identified CE as the third most prevalent foodborne parasitic disease worldwide [3]. Despite the WHO’s goal to control and ultimately eliminate the disease by 2050, the current global burden of CE prevention remains substantial, with annual control costs exceeding 3 billion United States Dollar (USD), of which China accounts for a significant proportion [2].

CE, caused by *Echinococcus granulosus* infection, primarily circulates between carnivores (definitive hosts) and herbivorous livestock (intermediate hosts), with humans typically serving as accidental hosts [4]. Human infection occurs when egg-contaminated water is accidentally ingested. The eggs hatch in the intestine, releasing oncospheres that disseminate via the portal vein or lymphatic system to various organs, primarily the liver [5, 6]. Pathologically, it manifests as progressively expanding cysts surrounded by fibrous pericyst layers [7]. Most patients remain asymptomatic during early stages, with clinical manifestations depending on cyst size, location, and complications [8]. Hepatic echinococcosis (in 80% of cases) progresses slowly, whereas pulmonary involvement (in 15% of cases) demonstrates more rapid progression [9]. Involvement of other organs presents greater diagnostic challenges and may result in severe complications [10]. CE is highly endemic in western China, Central Asia, and Africa [5]. Projections suggest China’s disease burden will continue escalating, with mortality rates reaching 4% in some underdeveloped regions [11]. CE threatens both human health and livestock industry development, exacerbating public health crises and economic disparities. Despite substantial control investments, China’s CE prevention outcomes lag behind international benchmarks [12].

Although research on CE in China has covered various areas, studies from a macro-comparative perspective remain insufficient. This gap obscures key issues: whether mortality declines are offset by rising infections, the quantified impact of demographic shifts, and the global relationship between sociodemographic development and CE burden. To address this, we analyzed GBD 2021 data (1990–2021) to: (1) compare long-term trends in CE incidence, prevalence, mortality, and DALYs in China and globally; (2) quantify demographic and epidemiological contributions to China’s burden via decomposition analysis; (3) examine the Sociodemographic Index (SDI)-burden association and China’s position; (4) benchmark China’s performance against the global efficiency frontier; and (5) identify age and sex-specific burden patterns. Our findings aim to optimize national control strategies and advance global CE elimination.

## Methods

### Study design

Data on CE for this study were sourced from the Global Burden of Diseases, Injuries, and Risk Factors Study (GBD) 2021 database. The GBD database provides comprehensive estimates based on six core metrics: incidence, mortality, prevalence, disability-adjusted life years (DALYs), years lived with disability (YLDs), and years of life lost (YLLs). These estimates are generated for various demographic strata, including age groups, sex, 204 countries and territories, and five SDI levels. SDI is a composite measure of educational attainment, income per capita, and total fertility rate [13]. The GBD database employs diverse data collection methods and undergoes regular updates [14]. Leveraging this resource, our study systematically assessed CE burden trends globally and within China from 1990 to 2021. This assessment specifically examined trends in incidence, prevalence, mortality, and DALYs, stratified by age and sex. The findings aim to provide an evidence base to inform policy formulation and enhance CE prevention strategies. As the GBD 2021 database is publicly accessible, this study adheres to the Guidelines for Accurate and Transparent Health Estimates Reporting (GATHER), ensuring methodological rigor and transparency.

### Data sources

CE-related data spanning 1990–2021 were retrieved from the GBD 2021 database (https://www.healthdata.org/research-analysis/gbd-data), including sex-stratified incidence, prevalence, mortality, and DALYs. To enable age structure-independent comparisons, we calculated age-standardized rates: age-standardized incidence rate (ASIR), age-standardized prevalence rate (ASPR), age-standardized mortality rate (ASMR), and age-standardized DALY rate (ASDR). Temporal trends were quantified using annual percentage change (APC) and average annual percentage change (AAPC), while crude rates, including the crude incidence rate (CIR), crude prevalence rate (CPR), crude mortality rate (CMR), and crude DALY rate (CDR), were computed to reflect the absolute burden magnitude. The incidence rate is defined as the number of new cases of a disease occurring in a defined population during a specified time period. It is typically expressed per 1000 or per 10,000 individuals and measures the risk of developing the disease. Prevalence (often point prevalence) is the proportion of individuals in a defined population who have a disease at a specific point in time. It is usually expressed per 1000 or per 10,000 persons and reflects the overall disease burden. The mortality rate (or cause-specific mortality rate) is the proportion of deaths in a defined population attributed to a specific cause over a given time period. It is used to assess the severity (or fatality) of a disease. DALYs were computed as the YLL and YLD, representing the standardized metric for quantifying total health loss from CE onset to mortality. YLL calculations incorporated age-specific CE mortality estimates and GBD reference life expectancy, while YLD estimations integrated CE prevalence with corresponding disability weights [15]. The analysis stratified individuals into distinct age groups.

### Statistical analysis

Within the GBD analytical framework, the 95% uncertainty intervals (UI) for all estimates were computed. Joinpoint regression software was utilized to calculate the AAPC and its 95% UI to identify temporal trends [16]. Log-transformed age-standardized rates (*y*) were fitted to a linear regression model:${\mathrm{ln}}\,{\mathrm{(y)}}\,{ = }\,{\upalpha }\,{ + }\,{\beta x}\,{ + }\,\varepsilon { }$ where *y* represents the age-standardized rate and *x* represents the calendar year, assuming a linear relationship between ln(y) and time. The AAPC was calculated as ${\text{AAPC }} = { }\left( {{\mathrm{exp}}\left( \beta \right){ } - { }1} \right){ } \times { }100$. Here, *β* is the regression coefficient representing the estimated annual average change in ln(*y*). The term exp(*β*) transforms the change back to the original rate ratio scale, and subtracting 1 followed by multiplication by 100 converts this ratio into a percentage change. The 95% confidence interval for the AAPC was derived by exponentiating the confidence interval for *β*: a trend was considered significantly increasing if the entire 95% UI was greater than 0, significantly decreasing if entirely less than 0, and nonsignificant if the 95% UI included 0. This study employed a decomposition analysis based on the Das Gupta method to quantify the drivers of the total change in DALYs between 1990 and 2021. The model constructed counterfactual scenarios to disentangle the total change in DALYs—without a residual term—into the independent contributions of three factors: population growth, changes in population age structure, and changes in age-specific epidemiological rates. The core of this approach lies in its stepwise factorization procedure, which sequentially holds two factors constant at either their base period (1990) or end period (2021) values, thereby isolating the net effect of the third factor. As a result, the overall change in DALYs is fully decomposed into the sum of effects attributable to population size, age structure, and epidemiological rates. The efficiency frontier, representing the minimum achievable ASDR for each level of the SDI, was constructed using a two-stage approach. First, the Free Disposal Hull (FDH) analysis was applied to identify best-performing countries and establish an initial frontier. This preliminary boundary was subsequently smoothed using locally estimated scatterplot smoothing (LOESS) regression. To ensure robustness, input values for smoothing were derived from 1000 bootstrapped mean ASDRs per SDI quintile. The effective gap—defined as the vertical distance between a country’s observed ASDR and the frontier value at its corresponding SDI—was used to quantify unrealized health gains achievable through alignment with top-performing peers at similar developmental levels. The efficiency gap is calculated as the difference between a country’s actual disease burden (ASDR) and the optimal performance frontier derived through bootstrap data envelopment analysis.

Statistical analysis and visualization were conducted using R software (version 4.4.2) and the Joinpoint Regression Program (version 5.3.0). A *P*-value of less than 0.05 was considered statistically significant. The GBD study has received approval from the Institutional Review Board (IRB) at the University of Washington [17].

## Results

### Global and Chinese CE disease burden trends: 1990–2021

Between 1990 and 2021, the number of new and prevalent cases of CE increased both globally and in China. The rise was particularly pronounced in China, where incident cases grew by 248% and prevalent cases by 285%, far exceeding the global increases of 64% and 70%, respectively. Despite the rising case numbers, mortality, and DALYs attributable to CE showed substantial declines worldwide. The number of global deaths fell by over 60%, while in China, the reduction was nearly 73%. This positive trend is further reflected in the age-standardized rates, underscoring improvements in disease management and treatment over the three decades (Table 1).

**Table 1:** In 1990 and 2021, cystic echinococcosis (CE) showed the following metrics across all age groups: incidence, prevalence, mortality, disability-adjusted life years (DALYs), age-standardized incidence rate (ASIR), age-standardized prevalence rate (ASPR), age-standardized mortality rate (ASMR), age-standardized DALY rate (ASDR), and average annual percentage change (AAPC)

Location	Measure	1990		2021		1990–2021 AAPC
		all-ages cases	Age-standardized rates per 100,000 people	all-ages cases	Age-standardized rates per 100,000 people	
		*n* (95% UI)	*n* (95% UI)	*n* (95% UI)	*n* (95% UI)	*n* (95% UI)
China	Incidence	7183 (5359–9582)	0.641 (0.483–0.851)	24,977 (18,714–32,390)	1.56 (1.178–2.044)	2.94 (2.72–3.17)
	Prevalence	29,499 (22,054–39667)	2.63 (1.988–3.487)	113,577 (85,533–145,961)	6.778 (5.132–8.896)	3.13 (2.90–3.36)
	Deaths	273 (232–317)	0.031 (0.027–0.036)	75 (60–93)	0.004 (0.003–0.005)	−6.36 (−6.56 to −6.15)
	DALYs	15,172 (12,939–17,942)	1.431 (1.22–1.679)	10,400 (6868–14,212)	0.615 (0.407–0.84)	−2.71 (−2.86 to −2.57)
Global	Incidence	90,312 (74,476–111,309)	1.762 (1.47–2.147)	148,521 (119,838–183,224)	1.822 (1.479–2.264)	0.12 (0.07–0.16)
	Prevalence	372,438 (306,424–466293)	7.368 (6.136–9.057)	633,404 (517,477–782,469)	7.691 (6.269–9.507)	0.14 (0.10–0.18)
	Deaths	3755 (2944–4569)	0.075 (0.06–0.092)	1364 (986–1775)	0.017 (0.012–0.022)	−4.68 (−4.76 to −4.60)
	DALYs	258,248 (205,098–325271)	4.675 (3.738–5.835)	105,072 (78,967–133,309)	1.32 (0.991–1.688)	−4.00 (−4.07 to −3.94)

Analysis of ASIR and ASPR reveals distinct trajectories. While global ASIR and ASPR saw minimal increases, China experienced sharp annual increases, with AAPC of 2.94% for incidence and 3.13% for prevalence. Conversely, China also demonstrated a much steeper decline in its age-standardized mortality rate, with an AAPC of–6.36%, highlighting significant advancements in medical interventions and outcomes compared with the global average (Table 1).

### Joinpoint regression analysis of the global and Chinese CE burden

**Fig. 1:**
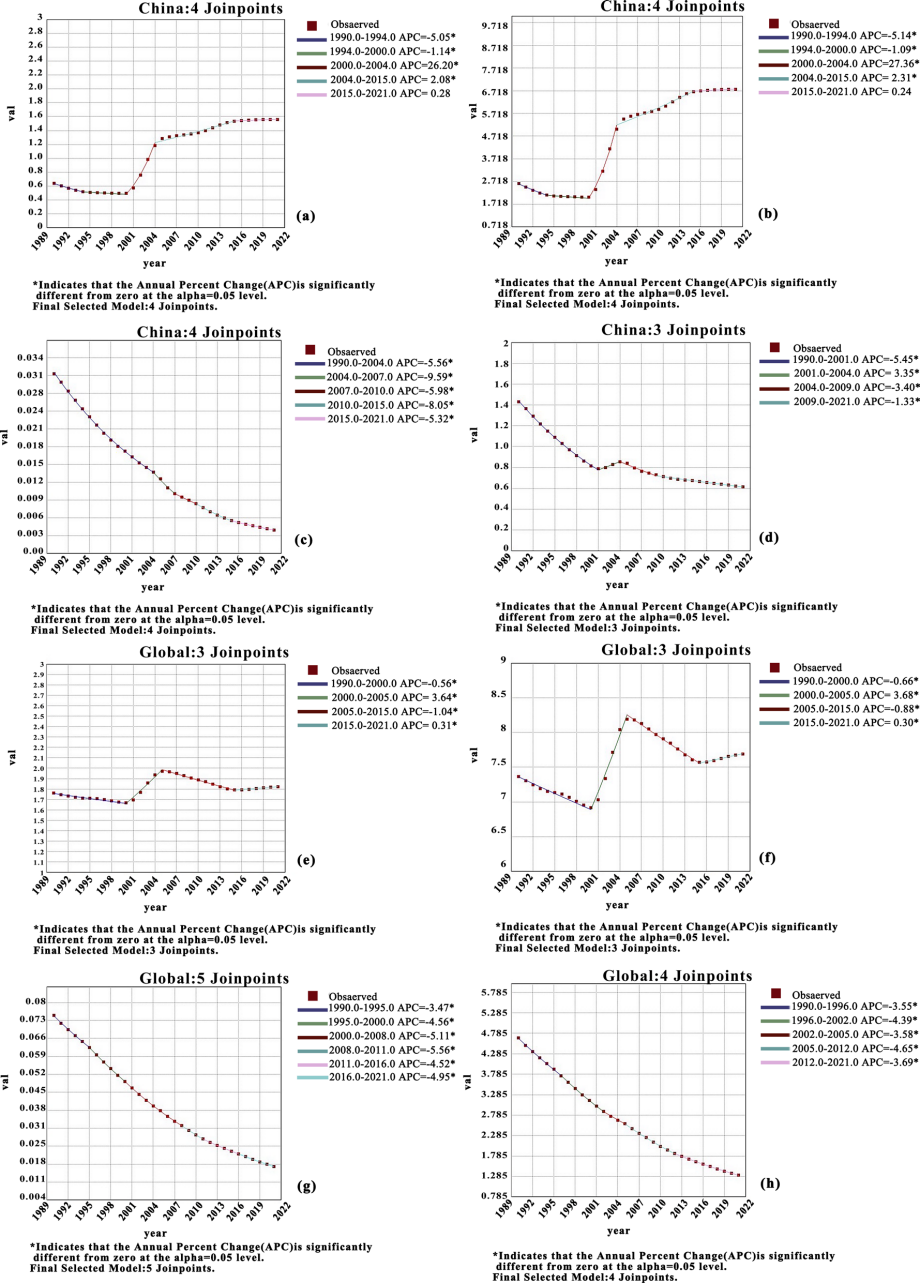
Annual percentage change (APC) of the age-standardized incidence rate (ASIR, **a**), age-standardized prevalence rate (ASPR, **b**), age-standardized mortality rate (ASMR, **c**), and age-standardized disability-adjusted life year rate (ASDR, **d**) for cystic echinococcosis (CE) in China, 1990–2021. APC of ASIR (**e**), ASPR (**f**), ASMR (**g**), and ASDR (**h**) for CE globally, 1990–2021. Asterisks (*) indicate statistically significant trends (P < 0.05)

In China, the ASIR and ASPR of CE exhibited a declining trend from 1990 to 2000 (ASIR: APC = −5.50 during 1990–1994 and −1.14 during 1994–2000, P < 0.05; ASPR: APC = −5.14 during 1990–1994 and −1.09 during 1994–2000, P < 0.05). However, over the next two decades, both rates showed a continuous increase, with the most significant rise occurring between 2000 and 2004 (ASIR: APC = 26.20, P < 0.05; ASPR: APC = 27.36, P < 0.05).In contrast, the ASMR and DALYs displayed minor fluctuations but an overall decreasing trend from 1990 to 2021 (Fig. 1a, b, c, d). Globally, the ASIR and ASPR of CE showed a significant increase during 2000–2005 (ASIR: APC = 3.64, P < 0.05; ASPR: APC = 3.68, P < 0.05), followed by slight fluctuations thereafter. From 1990 to 2021, the ASMR and DALYs demonstrated a consistent downward trend (Fig. 1e, f, g, h). During 1990–2021, both global and Chinese CE data revealed an upward trend in the APC of ASIR and ASPR, whereas the APC of ASMR and ASDR showed a declining trend (P < 0.05).

### Comparison of ASR burden trends for CE between the global level and China

**Fig. 2:**
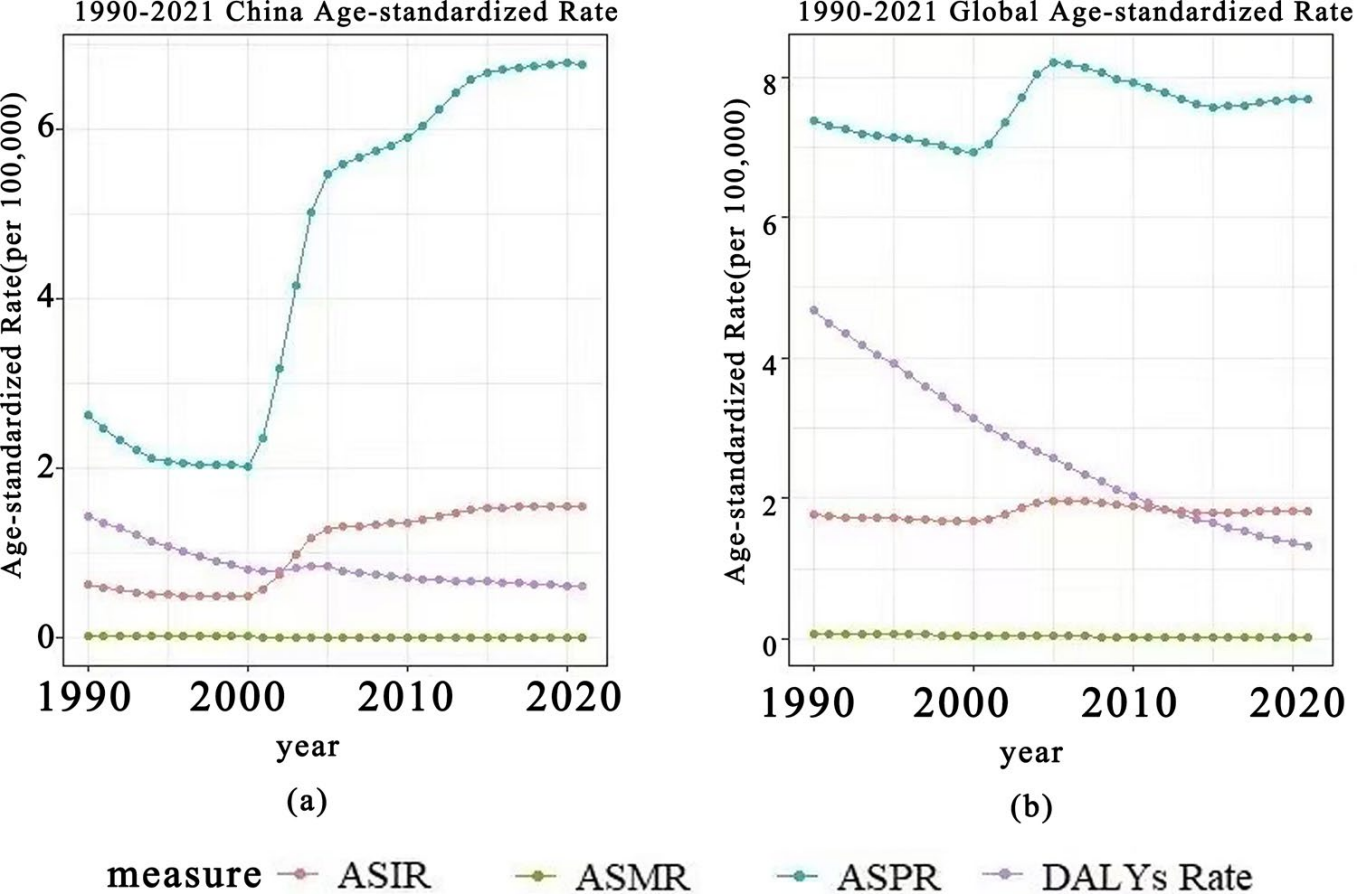
Age-standardized incidence rate (ASIR), age-standardized prevalence rate (ASPR), age-standardized mortality rate (ASMR), and age-standardized disability-adjusted life year rate (ASDR) of cystic echinococcosis (CE) in China (**a**) and globally (**b**), 1990–2021

From 1990 to 2021, the ASIR of CE in China was initially lower than the global level in 1990 but gradually increased after 2000, reaching a relatively stable level by 2005 (Fig. 2a). Globally, the ASIR was higher in 1990, showed a slight increase by 2000, and then remained relatively stable (Fig. 2b). In China, the ASPR was higher in 1990, rose sharply after 2000, and subsequently remained at an elevated level. This trend mirrored the pattern of ASIR, demonstrating a marked rise in case numbers. Globally, the ASPR was higher than that of China in 1990, increased again by 2000, peaked around 2005, then declined and stabilized thereafter. The ASIR and ASPR displayed an overall upward trend in the figures, whereas the ASMR remained relatively stable. Both the global and Chinese ASDRs were high in 1990 but exhibited a gradual decline over time. The trends in China and globally share some similarities but also show notable differences. Despite the rising number of cases, the decline in ASDR suggests a reduction in the disease’s health impact, likely attributable to advancements in medical technology and improved preventive measures. Future research should investigate the underlying causes of these trends further and explore more effective strategies for CE control and prevention (Fig. 2).

### Decomposition analysis of CE in China, 1990–2021

**Fig. 3:**
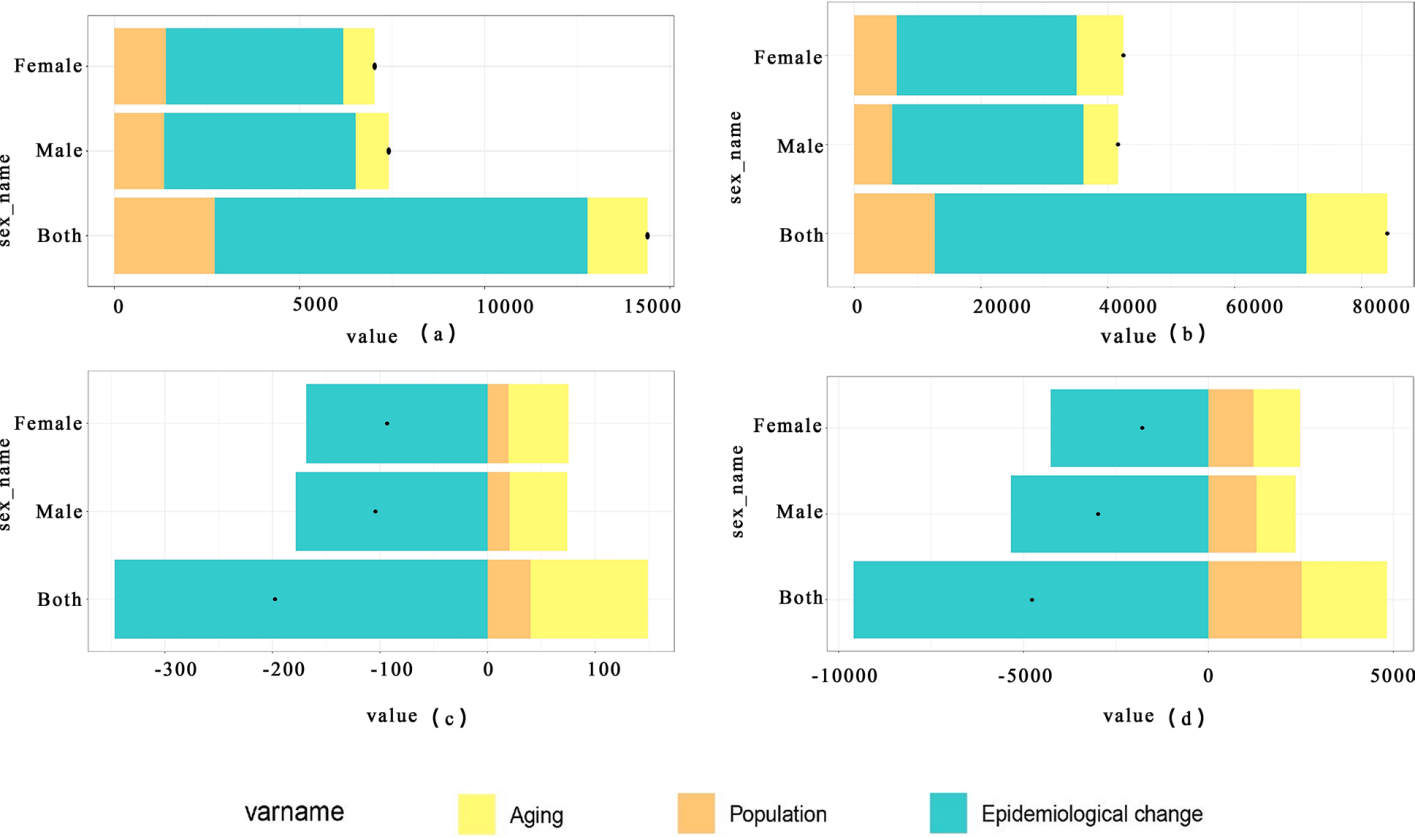
Contributions of population growth, aging, and epidemiological change to CE (**a**) incident cases, (**b**) prevalent cases, (**c**) death cases, and (**d**) disability-adjusted life years (DALYs) in China, for males, females, and both sexes combined, 1990–2021. The black dot represents the aggregate change attributable to all three components combined. For each component, the magnitude of positive values indicates the corresponding increase in burden attributed to that component, while the magnitude of negative values indicates the corresponding decrease in burden attributed to that component

As illustrated in Fig. 3, decomposition analysis revealed that epidemiological deterioration was the primary driver of the increase in both incidence and prevalence rates in China, accounting for 69.91% and 69.68% of the rise, respectively. Its impact far exceeded the combined effects of population growth and aging. This indicates a persistent deterioration in disease transmission risks, such as livestock infection, environmental contamination, and insufficient control measures. Concurrently, population growth exerted a greater influence on female incidence compared with males, potentially linked to pastoral women’s engagement in high-risk activities (e.g., feeding dogs, herding, milking, and handling feces). The heightened contribution of aging to prevalence (15.2%) reflects the chronic nature of CE and underscores the need for enhanced chronic disease management alongside ongoing control efforts. Conversely, the significant decline in mortality and DALYs was primarily driven by epidemiological improvements, likely attributable to breakthroughs in medical technology (e.g., advancements in surgical techniques, novel drug applications). This improvement was the decisive factor, accounting for 175.39% of the mortality decrease and 201.12% of the DALYs decrease, thereby completely offsetting the upward pressure on burden exerted by population growth and aging. Notably, females derived substantially greater benefit from these epidemiological improvements (238.59% contribution to mortality/DALYs decline versus 179.03% in males). However, they also bore a disproportionately heavier burden from aging (–70.52% contribution to increasing prevalence burden versus –35.43% in males), highlighting inadequacies in the management of elderly female patients.

### Stratified analysis of global multidimensional CE burden by SDI

China, alongside other high-SDI countries (e.g., Europe, North America, Japan, South Korea), exhibited ASIR and ASMR values below the threshold line, contrasting sharply with low-to-middle-SDI nations, particularly high-burden regions with SDI < 0.5. Within the high-middle SDI group, China’s ASPR ranked in the medium range. Although remaining below the threshold, it exceeded levels observed in most peer countries with comparable SDI. For ASDR, China was positioned in the low-middle range and below the threshold, yet within the high-SDI group, its ASDR was higher than that of most developed nations. High-burden countries were concentrated in low-SDI regions (SDI < 0.5), where all burden metrics, especially ASPR and ASDR, substantially exceeded the threshold. While all burden indicators in China fell below the threshold line, indicating it did not meet the high-burden criteria, its ASPR and ASDR were relatively elevated compared with countries with similar SDI levels. This likely reflects the residual impact of historical infection accumulation or persistent transmission.

Both ASIR and ASPR showed strong negative correlations with SDI. Low-SDI regions (for example, North Africa and the Middle East, Eastern Sub-Saharan Africa) exhibited significantly higher ASIR and ASPR, potentially attributable to poor sanitation, inadequate livestock management practices, and insufficient public health investment. In low-SDI areas, ASIR may decline slowly or remain persistently high. Conversely, in high-middle SDI regions (e.g., Southern Latin America, North Africa, and the Middle East), ASIR is expected to show a more pronounced downward trend with ongoing development (increasing SDI) and implementation of control measures. The decline in ASPR lagged behind that of ASIR, reflecting the historical accumulation of chronic infections. ASMR demonstrated the steepest negative correlation with SDI. Very low-SDI regions historically experienced high mortality, likely due to diagnostic delays and poor treatment access. However, ASMR has shown a significant declining trend, particularly in low-SDI areas, reflecting healthcare improvements. In high-SDI and some middle-SDI regions, ASMR is very low or approaches zero, indicating that well-established diagnostic capabilities and therapeutic interventions can largely prevent CE-related deaths. ASDR provides a more comprehensive quantification of the devastating health loss inflicted by CE as an NTD in low-SDI regions. In middle-SDI areas where ASMR has been somewhat controlled, the proportion of burden attributable to CE morbidity becomes more prominent. Understanding the spatiotemporal patterns across the dimensions of CE burden (incidence, prevalence, mortality, disability) at different SDI levels enables the precise identification of priority public health intervention areas at various stages of development, ultimately supporting progress toward the goal of CE elimination.

### Trends of CE burden by age groups in China, 1990–2021

As shown in Fig. 5, from 1990 to 2021, incident cases and prevalent cases of CE among Chinese patients, along with CIR and CPR, increased across all age groups, while deaths and CMR decreased. DALYs and CDR rose with age but declined in the > 60 age group. This suggests an overall increase in CE burden over 30 years. From 1990 to 2021, CE incident cases, CIR, prevalent cases, and CPR increased in all ages, most markedly in the 45–54 year group. The 45–54-year group had high values in both 1990 and 2021 (peak in 2021), likely linked to lifestyle/environmental factors. Other age groups showed lower but increasing trends, especially the 55–64 year and 65–74 year groups (Fig. 5a, b). In 1990, 0–14y had the highest deaths, DALYs, CMR, and CDR; in 2021, deaths increased with age (Fig. 5 c, d). Figure 5c–d indicates CE treatments achieved significant success, especially in reducing deaths/DALYs in children/adolescents. Despite progress, incidence/prevalence remains high in middle-aged groups (e.g., 45–54 years). Interventions needed: health education, vaccination, environmental monitoring, sanitation improvement, and animal host control.

### Analysis of gender-specific trends in CE burden in China from 1990 to 2021

As shown in Fig. 6, in both 1990 and 2021, the incidence and prevalence of CE in China were generally higher in females than in males, whereas the opposite pattern was observed for mortality and DALYs. In 2021, the number of incident cases among females aged over 30 years exceeded that of males, peaking in the 45–49 age group, followed by a gradual decline with advancing age (Fig. 6e). The gender disparity was most pronounced in the 35–39 age group, a pattern similarly observed in 1990, after which the gap gradually narrowed (Fig. 6a). A similar trend was noted for prevalence, with a higher number of affected females than males in 1990, and the most substantial gender disparity also occurred in the 35–39 age group (Fig. 6b). In 2021, age 35 served as a threshold: males under 35 had a slightly higher prevalence than females, whereas females over 35 significantly outnumbered males, with the largest disparity observed in the 65–69 age group (Fig. 6f). These variations may be attributed to age-specific physiological characteristics, lifestyle factors, and socioeconomic status changes among females. Although CE-related deaths have declined annually, male mortality rates consistently exceeded female rates (Fig. 6c and g). In 1990, CE-associated DALYs were higher in males under 65 years than in females, particularly in the 0–14 age group. By 2021, this pattern persisted, but with a marked reduction in DALYs for the 0–14 cohort (Fig. 6d and h). Despite higher incidence and prevalence in females, males exhibited greater mortality and DALYs, suggesting elevated case-fatality rates and disease burden post-CE infection among males. These gender disparities may stem from biological, behavioral, and socioeconomic determinants, warranting further investigation into why females are more susceptible to CE infection while males face higher postinfection mortality risks. In addition, disease burden escalates with age in females, whereas pediatric burden shows improvement.

Figure 7 demonstrates that ASIR and ASPR for both genders declined gradually from 1990 to 2000 but rose steadily over the subsequent 21 years. The most pronounced increase occurred during 2000–2005, with females exhibiting steeper upward trends in ASIR and ASPR than males, transitioning from slightly lower rates pre-2000 to surpassing males post-2005 (Fig. 7a and b). Overall, both genders showed ascending trends in incidence and prevalence (Fig. 7a, b, and 6). From 1990 to 2021, female deaths displayed a marked decline, accelerating post-2000. Males paralleled this trend with marginally steeper reductions, particularly after 2010 (Fig. 7c and 6). From 1990 to 2021, ASMR and ASDR remained higher in males. Although ASMR declined for both genders, females experienced a steadier reduction, whereas males showed slightly faster declines. Notably, post-2000 witnessed substantial ASDR reductions in males, accompanied by narrowing gender gaps in ASMR. ASDR for both genders also declined overall, despite a minor uptick during 2000–2005. Post-2005, male ASDR decreases accelerate, leading to progressively smaller gender disparities (Fig. 7c, d).

### Frontier analysis of the global CE Burden, 1990–2021

Figure 8a depicts the relationship between the ASDR for CE and the SDI globally from 1990 to 2021. Over time, ASDR generally decreased as SDI increased across countries, yet disparities in disease burden persisted among nations at different SDI levels. Countries with lower SDI typically experienced higher ASDR, while those with higher SDI exhibited lower rates, indicating that sociodemographic factors significantly influence the burden of CE. Figure 8b focuses on the change in CE ASDR relative to SDI globally in 2021 compared with 1990. Overall, substantial progress was made globally in reducing CE-related ASDR by 2021. Black labels identify the 15 countries with the largest gaps from the efficiency frontier, such as South Sudan and Tunisia, which continue to face significant challenges in disease control and remain considerably distant from the theoretical minimum burden. Blue labels highlight countries with low SDI but relatively favorable disease control outcomes, such as Haiti and Somalia, achieving notable success in controlling CE despite their low SDI. Dark red labels denote countries with high SDI but suboptimal control outcomes, exemplified by Lithuania and Norway, suggesting potential challenges in CE control even at advanced sociodemographic levels. Although China has been moving closer to the efficiency frontier, it has not yet fully achieved the theoretical minimum burden, indicating a persistent gap. This suggests that, given its current SDI level, China still has room for improvement in CE control and has not yet fully exploited its existing resources to achieve optimal prevention and treatment outcomes (Fig.8).

## Discussion

This study leveraged the GBD 2021 database to provide a comprehensive analysis of the CE burden in China from 1990 to 2021, situating its unique trajectory within the broader global context. Based on the AAPC of CE incidence and prevalence from 1990 to 2021 (AAPC for incidence in China: 2.94%, prevalence: 3.13%; global AAPC for incidence: 0.12%, prevalence: 0.14%), China exhibited a significantly increasing trend in both rates, far exceeding the global average. By 2021, China’s ASIR and ASPR of CE had increased by 143% and 158%, respectively, whereas the global ASIR and ASPR showed only marginal increases of 3.4% and 4.4% (Table 1). Joinpoint regression analysis (Fig. 1) further pinpointed the timing of this surge, identifying a dramatic upturn around 2000–2004 (APC for ASIR = 26.20%, ASPR = 27.36%), after a period of decline in the 1990s. This transition coincided with China’s implementation of the “Second National Survey on the Current Status of Key Human Parasitic Diseases” from 2001 to 2004, during which echinococcosis was formally incorporated into the national notifiable infectious disease reporting management system [18]. The establishment of this surveillance framework enabled more authentic and comprehensive documentation of previously underreported cases. This sharp reversal underscores a critical shift in the epidemiological landscape of CE in China post-2000. The decomposition analysis (Fig 3) revealed that epidemiological deterioration was the primary driver behind these trends. Numerous studies have emphasized the central role of biological factors in CE transmission [19]. For instance, in western China, the common practice of corearing dogs—definitive hosts of *Echinococcus granulosus*—with livestock leads to concentrated environmental contamination with canine feces, significantly increasing human infection risk [5]. The case of Tierra del Fuego, Chile, serves as a compelling illustration: a dog deworming program initiated in 1979 successfully reduced canine CE prevalence from 68.4% (1978) to 1.2% (2002). However, after the program was discontinued in 2004, surveillance in 2015–2016 revealed a sharp resurgence, with *Echinococcus* eggs detected in 45.4% of dog fecal samples [20]. This reversal underscores the critical importance of sustained intervention. Against this backdrop, China has effectively reduced CE incidence through integrated strategies including dog management, livestock vaccination, and community screening, with pilot projects in Sichuan and Tibet demonstrating particular success [21]. Nevertheless, CE epidemiology is complex, and canine infection levels do not always directly correlate with human incidence. A study in Tunisia exemplifies this: the region of Metlaoui had the highest canine egg contamination index (41.3%), yet it was not the area with the highest human incidence; in contrast, Kef, with a lower contamination rate (8.3%), reported higher human infection rates [22]. This suggests that sociobehavioral factors also play a key role. Specifically, in endemic areas such as Tunisia and western China, common local practices form critical transmission pathways: feeding untreated raw offal to domestic dogs during livestock slaughter, which perpetuates pathogen dissemination [23]; and consuming roadside barbecue from informally slaughtered and uninspected livestock, which directly increases human exposure risk [24]. Furthermore, the expansion of animal husbandry and live animal transport may facilitate the circulation of infected intermediate hosts, exacerbating disease spread [25]. Globally, notable regional disparities exist in CE control difficulty. A few island regions have achieved elimination, whereas mainland areas face greater challenges owing to host diversity, dietary practices, and governance complexities [26]. In China, this challenge is reflected in the geographic spread of cases to nonendemic areas. Surveillance data indicate that among 244 CE cases reported in nonendemic regions, 67.6% were imported (primarily from Xinjiang), while 23.8% were locally acquired [27]. This trend, driven by population mobility, increases the CE burden in nonendemic areas and complicates control efforts.

An epidemiological survey from 2012 to 2016 confirmed CE as a major public health threat in western China, with approximately 170,000 infected individuals and an estimated population at risk of around 50 million [28]. Despite rising case numbers over the past three decades, the ASMR attributable to CE has declined significantly—by 87% in China (from 0.031 to 0.004 per 100,000) and by 77% globally (from 0.075 to 0.017 per 100,000). This decline is largely attributed to overall improvements in epidemiological conditions (Fig. 3), particularly advancements in medical care and drug efficacy, which have offset the rising burden from population growth and aging. Although China achieved a faster reduction in ASMR than the global average (AAPC: –6.36%), the annual decline in DALYs lagged behind (AAPC: –2.71% versus global –4.00%) (Table 1). This paradox suggests that accumulating incident cases have partially offset treatment gains, highlighting the limitations of a control model emphasizing clinical treatment over prevention. The concurrent, more rapid global decline in both CE mortality (AAPC: –4.68%) and DALYs demonstrates that integrated strategies balancing prevention and treatment are more sustainable.

**Fig. 4:**
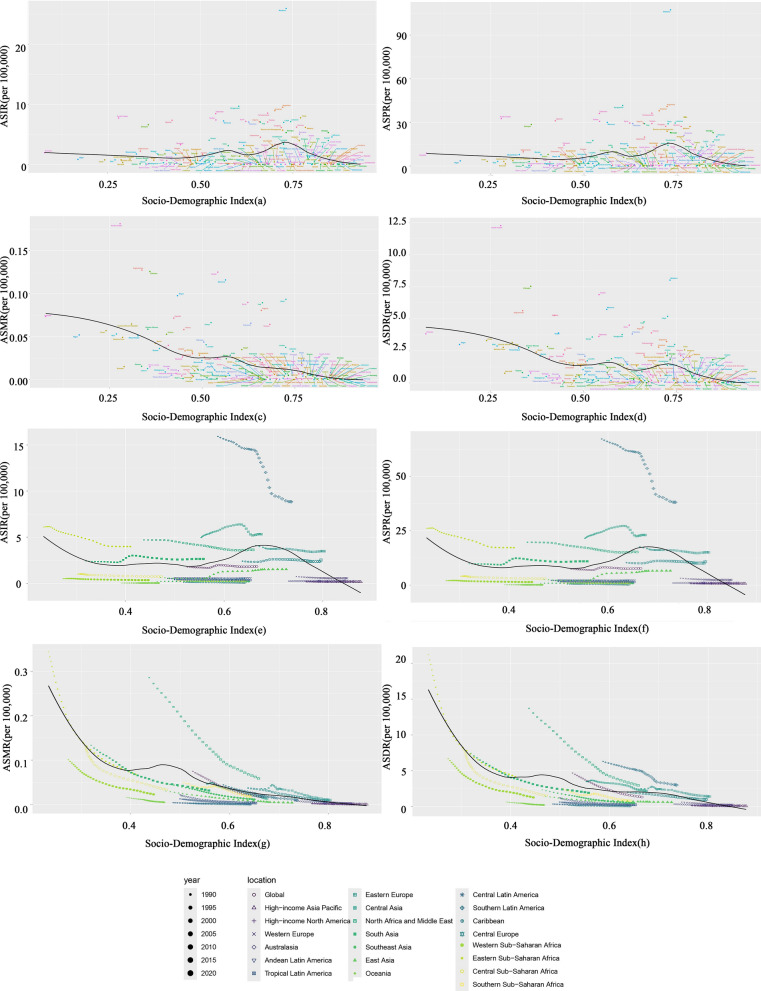
Association between the Sociodemographic Index (SDI) and CE burden per 100,000 population in 201 countries, 2021: (**a**) ASIR, (**b**) ASPR, (**c**) ASMR, (**d**) ASDR. Trends in CE burden per 100,000 population stratified by SDI level across 21 Global Burden of Diseases (GBD) super-regions, 1990–2021: (**e**) ASIR, (**f**) ASPR, (**g**) ASMR, (**h**) ASDR. The threshold line represents an objective cutoff distinguishing high-burden from low-burden countries/regions, aiding in the identification of priority public health intervention areas

The stratified analysis by SDI (Fig. 4) provides critical macro-level context, demonstrating a strong negative correlation between the global CE burden (ASIR, ASPR, ASMR, ASDR) and SDI, with low-SDI countries bearing the heaviest burden. The SDI—encompassing education, income, and fertility rate—significantly influences CE incidence and mortality. Yang et al. projected the global number of CE cases may reach 235,628 by 2030, with higher incidence in lower-SDI countries [29]. Furthermore, peak CE prevalence occurs at older ages in lower-SDI regions [30]. Consistent with our findings (Fig. 4 and 8), both ASIR and ASPR show significant negative correlations with SDI. Poor sanitation, inadequate livestock management, and insufficient public health investment in low-SDI areas likely contribute. Education and income levels influence occupations and lifestyles, while limited education may be associated with risky behaviors such as consuming untreated water or raw vegetables, and neglecting handwashing [28]. In the SDI frontier analysis, China is classified in the middle-high SDI group (Fig. 8). While China’s burden indicators remain below the high-burden threshold, its ASPR and ASDR are relatively high compared with peers with similar SDI levels, and it has not achieved the theoretical minimum burden attainable. This indicates China has not fully leveraged its resources for optimal disease control, suggesting a persistent efficiency gap. Therefore, alongside balancing prevention and treatment, China must strengthen dietary and health education, improve economic and educational conditions, and enhance performance across all SDI dimensions to reduce high-risk exposure.

**Fig. 5:**
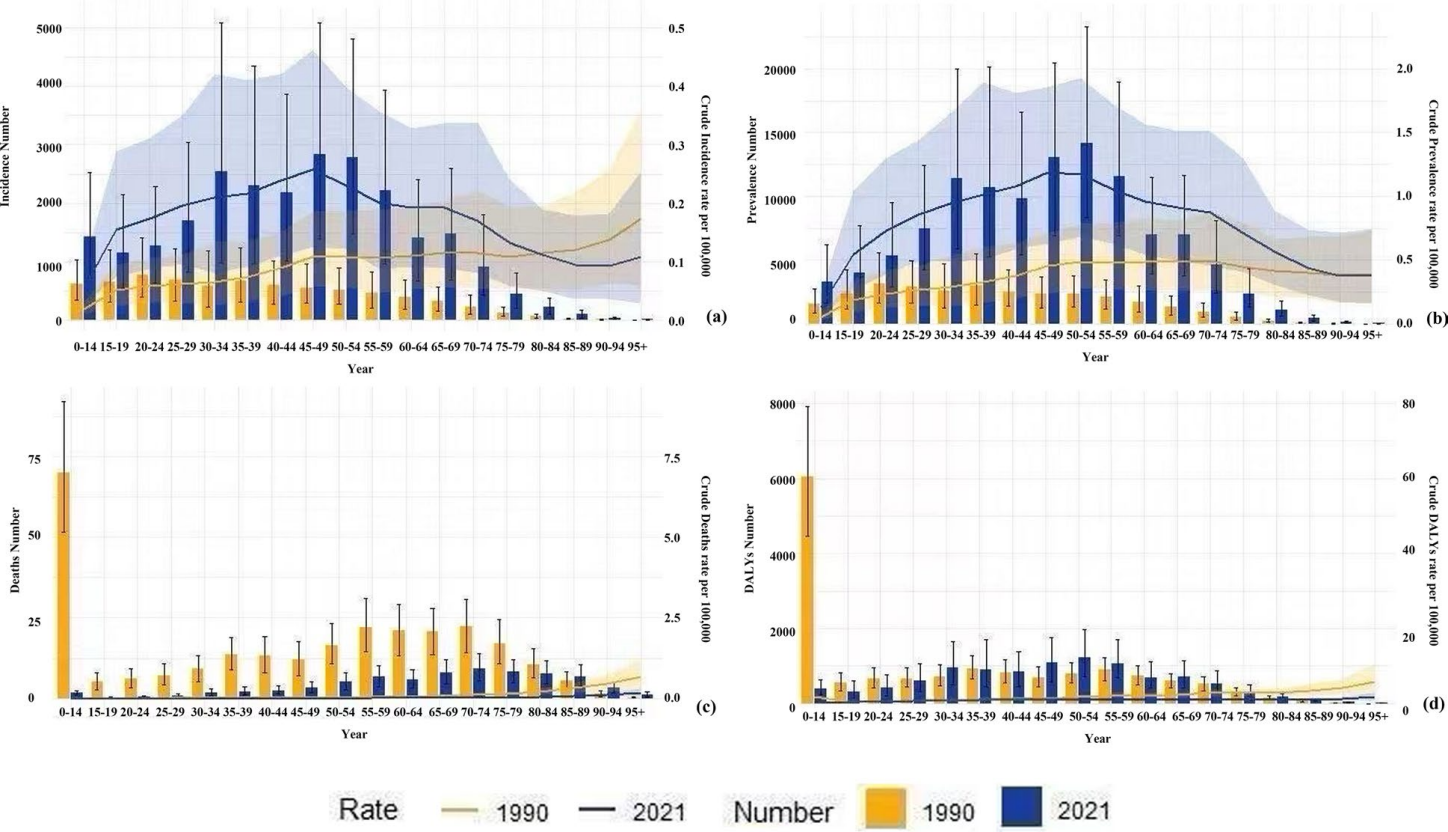
CE in China (1990–2021): age-specific patterns of (**a**) incident cases and crude incidence rate (CIR), (**b**) prevalent cases and crude prevalence rate (CPR), (**c**) death cases and crude mortality rate (CMR), and (**d**) DALYs and crude DALY rate (CDR) across different age groups

From 1990 to 2021, the CE burden in China showed significant age heterogeneity (Fig. 5). Among children and adolescents aged 0–14, mortality and disability rates decreased markedly, attributable to widespread vaccination, health education, and improved early diagnosis/treatment [31]. In contrast, middle-aged individuals (45–54 years) experienced the most rapid increase in incidence and prevalence, constituting the core burden. This aligns with CE’s long latent period [32]; early subclinical infections may only manifest 5–20 years postexposure [33]. Thus, the high burden in the 45–54 year old group likely stems from occupational exposure during their youth in contaminated environments [24]. Among older adults (> 60), mortality declined with age, but prevalence rose, reflecting the cumulative effect of past infections. Decomposition analysis (Fig. 3) highlighted the pronounced contribution of population aging to rising prevalence, underscoring CE’s chronic nature. Global demographic projections indicate the population aged > 60 will increase from 1 billion in 2020 to 1.4 billion by 2030 [34], suggesting aging combined with rising prevalence may exacerbate the future burden. Control strategies should be precisely targeted: for children, focus on risk prevention (e.g., dog deworming, environmental interventions); for middle-aged and older populations, adopt differentiated interventions such as long-term follow-up (e.g., > 5 years regular review) [35] to monitor progression and reduce recurrence.

**Fig. 6:**
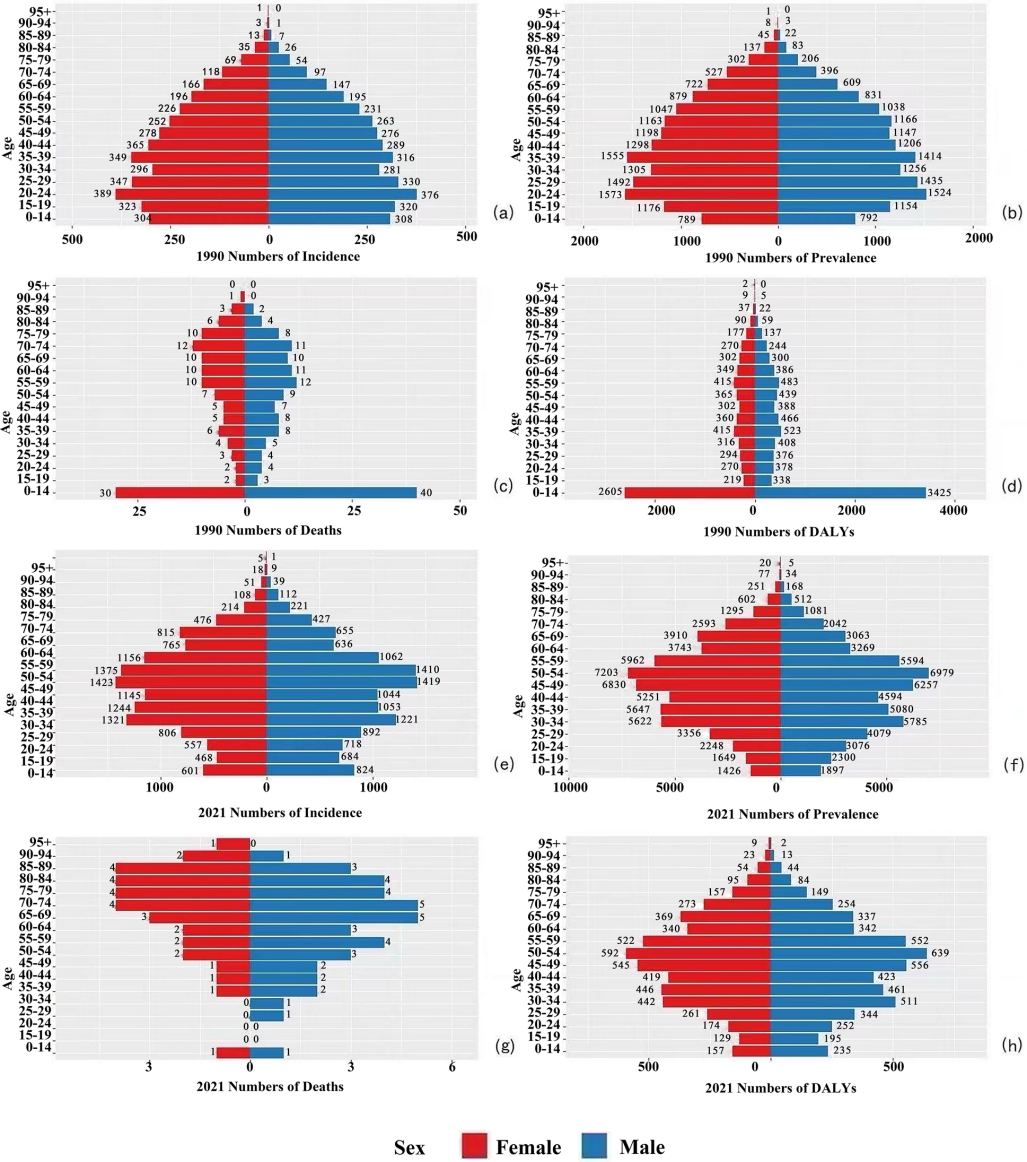
CE in China by age and sex (1990): (**a**) incident cases, (**b**) prevalent cases, (**c**) deaths, (**d**) DALYs. CE in China by age and sex (2021): (**e**) incident cases, (**f**) prevalent cases, (**g**) deaths, (**h**) DALYs

**Fig. 7:**
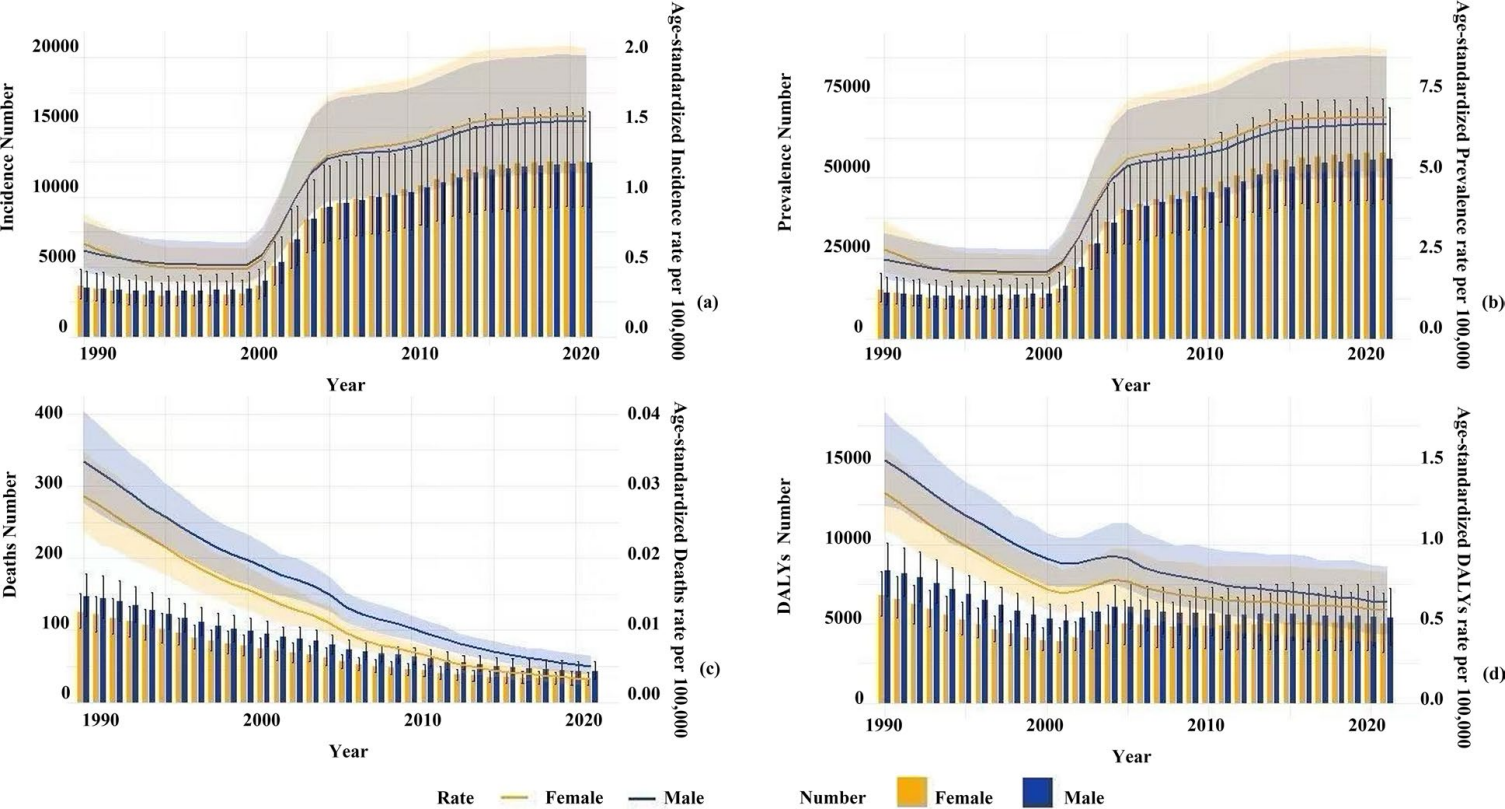
Temporal trends of CE in China by sex, 1990–2021: (**a**) annual incident cases with ASIR, (**b**) annual prevalent cases with ASPR, (**c**) annual deaths with ASMR, (**d**) annual DALYs with ASDR

Analysis of gender differences (Fig. 6 and 7) reveals a noteworthy paradox: women exhibit higher incidence and prevalence rates than men, particularly after age 35, yet male patients bear a significantly greater mortality and DALY burden. A survey in western China indicated women have a 1.35-fold higher infection risk [28], potentially linked to greater involvement in activities including feeding dogs, herding, and collecting feces, increasing exposure to *Echinococcus* eggs [36]. Occupational exposure is a significant route of parasitic infection [37]. Trend analysis (Fig. 7) suggests the widening gap in incidence/prevalence after 2000 may reflect increased female participation in livestock farming. Although women have higher infection rates, men experience worse outcomes, suggesting sex-based differences in immunopathology. Studies indicate that higher testosterone levels in men may suppress protective Th1 immune responses, impairing parasite clearance. In contrast, nonpregnant women, with higher estrogen levels, exhibit enhanced Th1 responses and macrophage activity, leading to better clinical outcomes [38]. Thus, although social gender roles place women at a higher exposure risk, their innate immune advantages partially mitigate the severity of infection. Based on these findings, further investigation into the mechanisms underlying age- and gender-specific differences in CE burden is warranted. Tailored public health strategies should be implemented: for women, emphasis should be placed on early screening (e.g., promoting annual ultrasound examinations in pastoral areas), while for men, efforts should focus on optimizing severe case management and long-term disease care.

**Fig. 8:**
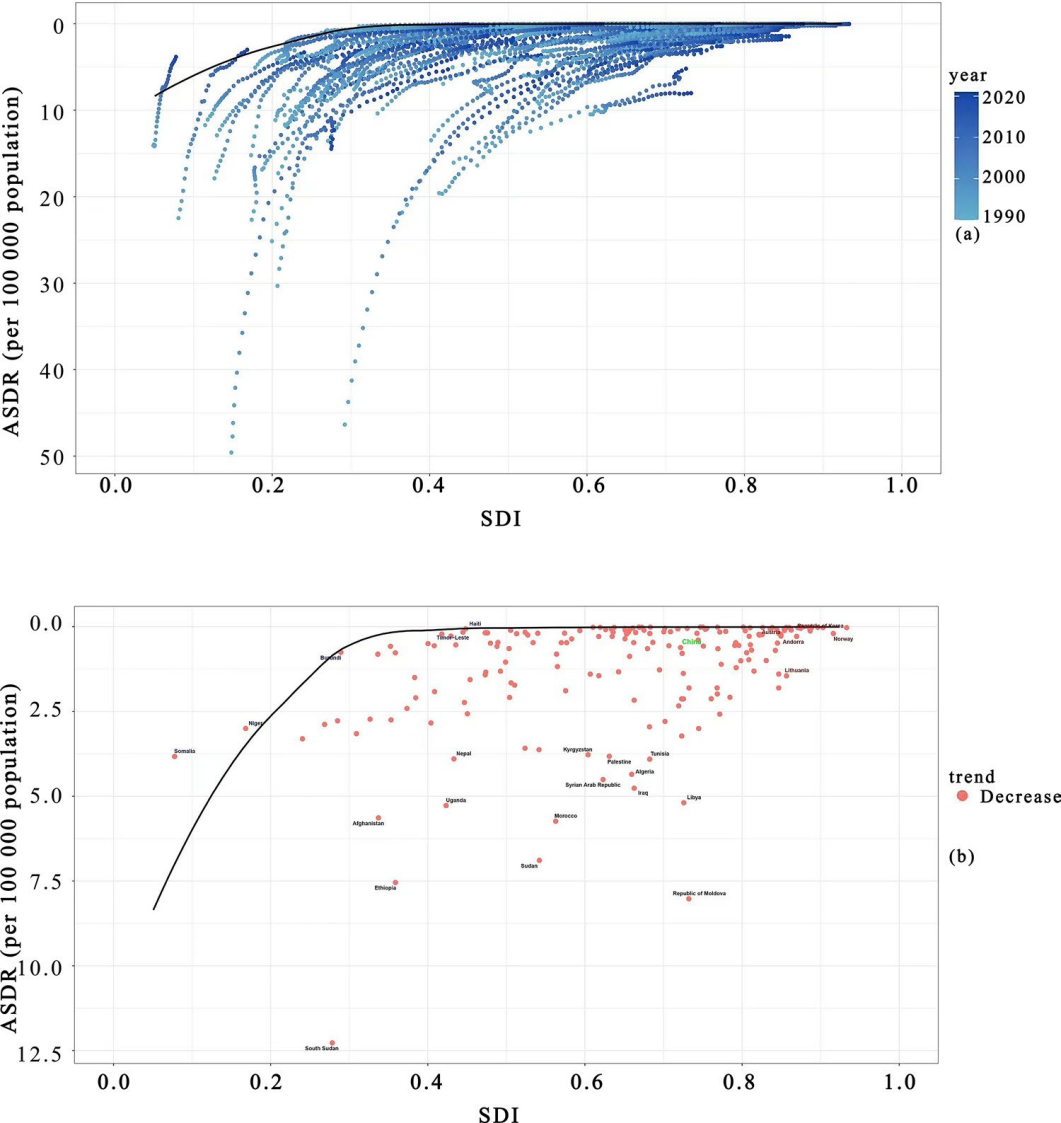
Globally, the relationship between the age-standardized disability-adjusted life year rate (ASDR) for CE and the Sociodemographic Index (SDI) from 1990 to 2021 is shown in panel **a**. This features the efficiency frontier (the theoretical minimum disease burden achievable at each SDI level) as a solid black line, with country data points colored by year (pale blue for 1990 progressing to dark blue for 2021). Panel **b** illustrates the change in this ASDR–SDI relationship between 1990 and 2021. Countries are categorized and labeled: black labels identify the 15 nations with the largest gap from the efficiency frontier (e.g., South Sudan, Tunisia); blue labels mark low-SDI (< 0.5) countries achieving high efficiency (e.g., Haiti, Somalia); dark red labels highlight high-SDI countries performing with low efficiency (e.g., Lithuania, Norway). China is distinctly highlighted with a green label. Light red points signify countries where the burden decreased in 2021 relative to 1990

The continuous decline in CE ASMR and ASDR globally and in China aligns with the WHO global NTD strategic framework. The Global Roadmap for NTDs (2012–2020) first defined control/elimination targets for 17 NTDs, emphasizing large-scale drug administration and multisectoral collaboration [39]. The 2012 London Declaration promoted multi-stakeholder cooperation [40]. The updated 2021–2030 roadmap sets specific targets for reducing incidence, prevalence, DALYs, and mortality by 2030 [41]. WHO-led initiatives have collectively contributed to reducing the global and Chinese CE burden. Within this context, China’s CE control pathway exhibits a unique phased evolution: passive control (pre-1980), pilot control (1980–2004), integrated management (2005–present), and precision control (starting 2023) [18]. This progression reflects a transition from fragmented interventions to a systematic, nationwide strategy and toward data-driven precision control. Investments in population screening and free treatment between 2005 and 2010, and the implementation of action plans, expanded control efforts [26]. The release of the National Integrated Management Plan for Echinococcosis and Other Key Parasitic Diseases (2024–2030) marks China’s entry into a new precision control phase aimed at elimination, forming a distinctive “Chinese model.” However, frontier analysis (Fig. 8) indicates China, while approaching the global efficiency frontier, has not achieved the theoretical minimum burden for its SDI, reflecting an “efficiency gap.” Future efforts must break through technical bottlenecks in diagnostics, drug/vaccine research, while strengthening intelligent surveillance, regional collaboration, and multidisciplinary integration to advance elimination and provide scalable solutions for global CE control.

Several limitations should be acknowledged. First, the analysis relies on GBD estimates, which may be affected by data quality and underreporting. Second, while the study examines temporal trends and conducts decomposition analysis, the lack of access to primary clinical or environmental exposure data limits causal inference regarding specific risk factors, including livestock infection rates or environmental contamination. Third, the sex- and age-specific findings are based on aggregate-level estimates, which may obscure intra-group heterogeneity and prevent more granular risk stratification. Finally, frontier and SDI analyses reflect associations rather than causality, and may not fully account for local health system capacity, policy implementation efficiency, or sociocultural determinants.

## Conclusions

This study reveals a significant negative correlation between the global burden of CE and the SDI, with low-SDI countries bearing the majority of the disease burden. In China, the increase in incidence and prevalence is primarily driven by adverse epidemiological factors. Although the mortality rate has declined, the slower reduction in DALYs underscores the persistent challenges in the clinical management of CE. Middle-aged adults continue to be at risk of latent infection, while cumulative exposure contributes to the rising prevalence among the elderly. A notable gender paradox was also observed: although women exhibit a higher infection rate, men experience greater mortality and overall disease burden. Previous studies have confirmed that the prognosis of parasitic infections is associated with hormonal influences. Despite China being classified in the middle-high SDI group, its disease burden metrics remain below the high-threshold level. The age-standardized prevalence rate and age-standardized death rate have not yet achieved the theoretically attainable optimal control outcomes for their current SDI level. Addressing these disparities through targeted interventions for high-risk populations is essential for advancing toward the World Health Organization’s 2030 NTD control targets.

## Data Availability

The datasets generated during and/or analyzed during the current study are available from the Global Health Data Exchange query tool (http://ghdx.healthdata.org/gbd-results-tool).

## Abbreviations

WHO: World Health Organization
GBD: Global burden of diseases
GATHER: Guidelines for accurate and transparent health estimates reporting
FAO: United Nations food and agriculture organization
IRB: Institutional review board
NTD: Neglected tropical diseases
UI: Uncertainty intervals
USD: United States Dollar
FDH: Free disposal hull
LOESS: Locally estimated scatterplot smoothing
SDI: Sociodemographic index
CE: Cystic echinococcosis
APC: Annual percentage change
AAPC: Average annual percentage change
DALYs: Disability-adjusted life years
YLDs: Years lived with disability
YLLs: Years of life lost
ASIR: Age-standardized incidence rate
ASPR: Age-standardized prevalence rate
ASMR: Age-standardized mortality rate
ASDR: Age-standardized DALY rate
CIR: Crude incidence rate
CPR: Crude prevalence rate
CMR: Crude mortality rate
CDR: Crude DALY rate
